# High-efficiency nonlinear frequency conversion enabled by optimizing the ferroelectric domain structure in *x*-cut LNOI ridge waveguide

**DOI:** 10.1515/nanoph-2024-0168

**Published:** 2024-05-29

**Authors:** Yawen Su, Xinyu Zhang, Haiwei Chen, Shifeng Li, Jianan Ma, Wei Li, Yunfei Niu, Qi Qin, Shaoguang Yang, Yu Deng, Yong Zhang, Xiaopeng Hu, Shining Zhu

**Affiliations:** National Laboratory of Solid State Microstructures, College of Engineering and Applied Sciences, School of Physics, and Collaborative Innovation Center of Advanced Microstructures, 12581 Nanjing University, Nanjing 210093, China; College of Integrated Circuit Science and Engineering, National and Local Joint Engineering Laboratory of RF Integration and Micro-Assembly Technology, 12581 Nanjing University of Posts and Telecommunications, Nanjing 210023, China; 559075 Zhejiang Lab, Hangzhou 311121, China; State Key Laboratory of Radio Frequency Heterogeneous Integration, Key Laboratory of Intelligent Optical Measurement and Detection of Shenzhen, 47890 Shenzhen University, Shenzhen 518060, China; Quantum Science Center of Guangdong-Hong Kong-Macao Greater Bay Area (Guangdong), Shenzhen 518060, China

**Keywords:** lithium niobate on insulator, ferroelectric domain engineering, electric field poling, second harmonic generation

## Abstract

Photonic devices based on ferroelectric domain engineering in thin film lithium niobate are key components for both classical and quantum information processing. Periodic poling of ridge waveguide can avoid the selective etching effect of lithium niobate, however, the fabrication of high-quality ferroelectric domain is still a challenge. In this work, we optimized the applied electric field distribution, and rectangular inverted domain structure was obtained in the ridge waveguide which is beneficial for efficient nonlinear frequency conversions. Second harmonic confocal microscope, piezoresponse force microscopy, and chemical selective etching were used to characterize the inverted domain in the ridge waveguide. In addition, the performance of nonlinear frequency conversion of the periodically poled nano-waveguide was investigated through second harmonic generation, and the normalized conversion efficiency was measured to be 1,720 % W^−1^ cm^−2^, which is close to 60 % that of the theoretical value. The fabrication technique described in this work will pave the way for the development of high-efficiency, low-loss lithium niobate nonlinear photonic devices.

## 1 Introduction

In recent years, with the commercialization of thin film lithium niobate wafers and the development of ultra-low loss waveguide fabrication techniques [1], [2], lithium niobate on insulator (LNOI) has emerged as a promising material platform for integrated photonics [3], [4], [5], [6]. Due to the excellent properties of lithium niobate, such as low material loss, a large transparent window ranging from ultra-violet to mid-infrared, large electro-optic effect, and strong second-order optical nonlinearity, diverse photonic integrated circuits and devices have been realized based on LNOI platform [7]. Relevant applications include high-speed electro-optic modulators [8], [9], [10], [11], [12], compact acousto-optic devices [13], [14], [15], and ultra-efficient nonlinear frequency conversions [16], [17], [18]. Among the various photonic devices, nonlinear integrated photonic devices based on ferroelectric domain engineering have attracted great research interest for applications in both classical and quantum information processing [19], [20], [21]. Owing to the quasi-phase-matching technique, the strong light field confinement in the nano-waveguide, as well as the flexible modal dispersion engineering, highly efficient and low-power consumption second harmonic generation (SHG) [22], [23], sum frequency generation [24], [25], [26], third harmonic generation [27], optical parametric oscillator [28], [29], [30], multioctave supercontinuum generation [31], [32], [33], and high-brightness entangled photon pair generations [34], [35], [36], have been developed and intensively studied. To realize quasi-phase-matching in LNOI, two fabrication processes are essential, which are waveguide etching and ferroelectric domain inversion [37], [38], [39]. As is well known, periodically poled lithium niobate exhibits selective etching issues in reactive ion etching, and other processes such as chemical cleaning and chemical mechanical polishing, i.e. there is difference in the etching rate between the oppositely poled lithium niobate ferroelectric domains. Therefore, it is crucial to consider the process sequence for waveguide etching and domain inversion. The commonly used procedure is periodical domain inversion followed by waveguide etching, which will introduce sidewall roughness and subsequent extra propagation loss to the waveguides. An alternative approach to address the sidewall roughness caused by selective etching is to perform periodic poling after waveguide etching. In a previous work, local periodic poling of LNOI ridge waveguide was reported, and the normalized SHG conversion efficiency is only one tenth that of the theoretical value, which is mainly attributed to the non-ideal triangular domain shape [40].

The efficiency of nonlinear frequency conversion in periodically poled LNOI (PPLNOI) is related to the following factors: the effective second-order nonlinear coefficient, the spatial mode overlapping integral between the fundamental and harmonic optical fields, the wave-vector mismatch, and the shape of the inverted ferroelectric domain. To improve the nonlinear conversion efficiency, optimizing the inverted ferroelectric domain to the normal rectangular shape is crucial. In this paper, a comb-to-comb shaped electrode is specially designed and placed on both sides of the *x*-cut LNOI ridge waveguide, to provide a suitable external electric field distribution for ferroelectric domain inversion in the ridge waveguide. Through the high-voltage electric field poling technique [41], [42], nearly rectangular periodic domain inversion was achieved in the LNOI ridge waveguide. The inverted domain was revealed through second harmonic (SH) confocal microscopy, piezoresponse force microscopy (PFM) and selective chemical etching [43]. In addition, continuous-wave SHG in the fabricated sample was characterized, and the normalized conversion efficiency at 1,593 nm was measured to be 1,720 % W^−1^ cm^−2^, which is nearly 60 % that of the theoretical value.

## 2 Design and fabrication

**Figure 1:**
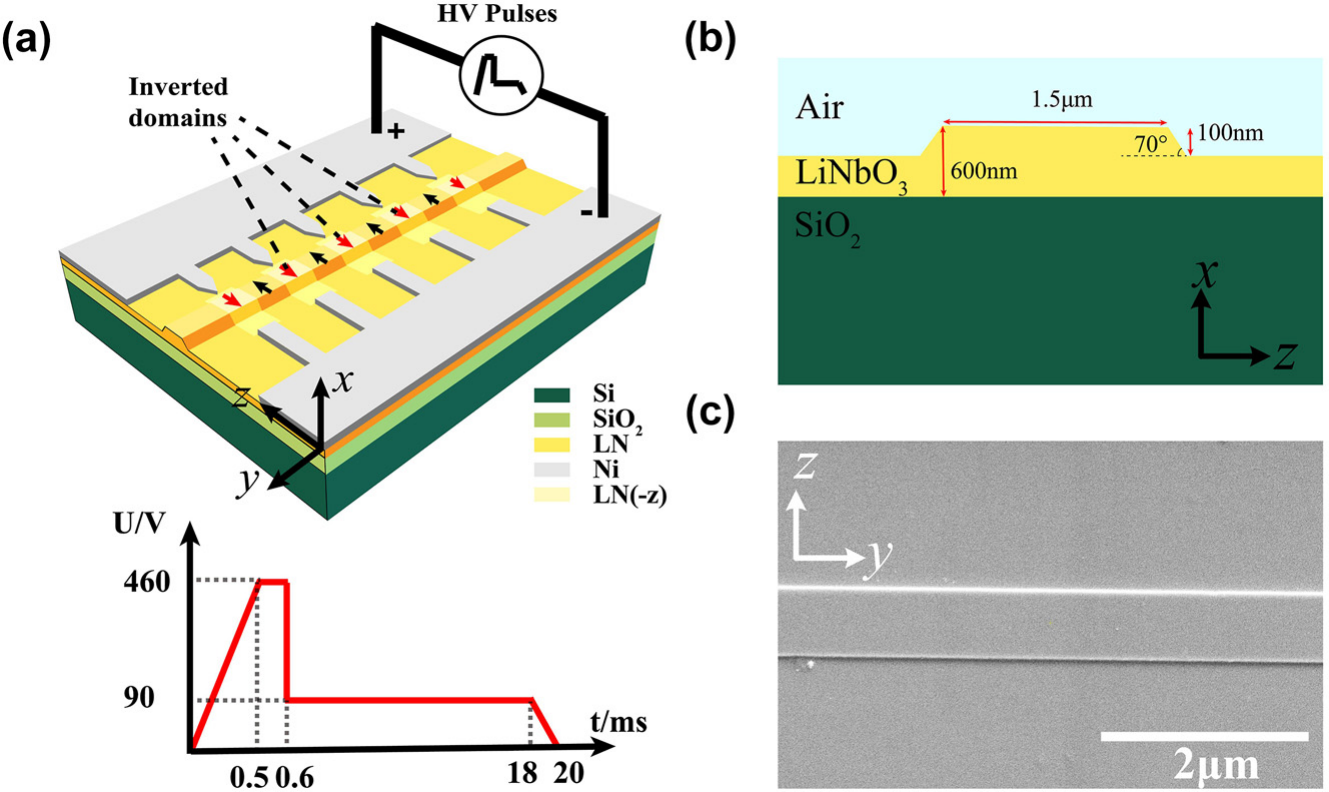
Images of the nonlinear waveguide. (a) Schematic diagram of electric field poling of ridge waveguide. High-voltage pulses are applied along the *z*-axis of the *x*-cut LNOI wafer. The lower left section is the schematic pulse shape. (b) Schematic geometry of the ridge waveguide, featuring a lithium niobate thin film with a thickness of 600 nm, an etched depth of 100 nm, and a width of 1.5 μm. (c) The scanning electronic microscope image of the LNOI nanophotonic waveguide after periodic poling.

The periodically poled ridge waveguide was fabricated on a 1.5 cm × 1.5 cm *x*-cut LNOI wafer (NANOLN Inc.). The thickness of the single-crystalline thin film is 600 nm, and the thin film was bonded to a 2 μm-thick thermally-grown silicon dioxide layer on a silicon substrate. The fabrication processes are described in the following: Firstly, metal alignment marks consisting of 30-nm Cr and 70-nm Au were deposited on the 600-nm-thick *x*-cut LNOI using UV lithography and electron beam evaporation. These marks were essential for aligning subsequent fabrication steps involving the etched ridge waveguide and the patterned electrode for periodic poling. Secondly, the desired areas for the ridge waveguides on the surface were defined using electron beam lithography (EBL). Ion beam etching was then exploited to dry etch the waveguide structure, followed by a cleaning process using RCA (7:1:2, deionized water, ammonia and hydrogen peroxide) solution to reduce the waveguide losses. Thirdly, 100-nm-thick comb-to-comb shaped nickel electrodes were defined on both sides of the waveguide using EBL and lift-off process. The tip of the electrode connecting to the positive terminal is a triangular one, while a flat tip is used for the electrode connecting to the negative terminal, and the waveguide is positioned between the two electrode teeth, as shown in Figure 1(a). The dimensions of the triangular electrode teeth are as follows: the rectangular section measures 20 µm in length and 1 µm in width, with a triangular tip extending 5 µm. The length of the rectangular negative electrode is 25 µm. The fourth step is, 28 high-voltage (HV) pulses, the repetition period being 4 s, were applied to obtain inverted ferroelectric domain structures in the ridge waveguides. The periodic poling procedure was executed in a controlled clean room environment, maintaining a temperature of 23.0 ± 1.0 °C and humidity levels around 50 %. The HV pulse shape is illustrated in the lower left section of Figure 1(a). The peak voltage is set at 460 V for a duration of 0.1 ms. Subsequently, a 90-V low voltage is maintained for 17.4 ms to prevent depolarization. The polarization of a single waveguide typically requires approximately 2 min to complete. Finally, the electrodes were removed using dilute hydrochloric acid, and the end faces of the waveguide were polished for optical measurements. The schematic geometry of the ridge waveguide is shown in Figure 1(b). The top width of the ridge waveguide was 1.5 μm and the etched depth was 100 nm, with the sidewall angle of the waveguide being 70°. Figure 1(c) is the scanning electronic microscope (SEM) image of the ridge waveguide after periodic poling, and the sidewall roughness caused by selective etching of anti-parallel ferroelectric domains is not observed. Using the Fabry–Perot method [44], the transmission losses of the waveguides were measured to be 0.23 and 0.25 dB/cm before and after the periodic poling process, correspondingly.

To realize high-quality ferroelectric domain inversion in LNOI ridge waveguides by applying high-voltage electric field, the choice of a proper electric field distribution is essential. In the previous work [40], a comb-to-pad electrode was attached to the sidewall of the ridge waveguide for periodic poling, and triangular ferroelectric domain was obtained. The formation of triangular domain was mainly attributed to the following: the electric field distribution differs a lot in profile and intensity at the positive and negative electrode regions due to the asymmetrical comb-to-pad electrode shape and the small spacing between the comb and pad, and thus a large lateral domain expansion occurred at the positive electrode. In order to optimize the inverted ferroelectric domain to the ideal rectangular shape, this study utilized a comb-to-comb electrode configuration. These electrodes are positioned away from the waveguide sidewall and at a distance from the ridge. Two key factors were identified as crucial for achieving rectangular domain reversal regions. Firstly, the electric field intensity within the domain reversal region primarily aligns along the *z*-axis, with minimal components in the *x* and *y* directions. This rationale guided the placement of electrodes at a specific distance from the ridge waveguide, as the electrode tips exhibit significant *x*/*y*-directional electric field components. Secondly, effective control of domain growth in the lateral direction is essential. The rapid and uniform lateral growth of domains is the primary cause of non-rectangular domains, often appearing as trapezoidal or triangular shapes. By employing needle-like electrodes and specific waveform characteristics of electrical pulses, better control of lateral domain growth during the reversal process is achieved, resulting in the realization of rectangular domain reversal regions. It should be noted that we designed the electrodes near the +*Z* direction to be needle-like because electrodes of this morphology have only one tip, in contrast to rectangular electrodes that have two tips. This implies that compared to needle-like electrodes, rectangular electrodes would lead to more pronounced lateral expansion of domains, which is undesired in the preparation of rectangular domain reversal structures. On the other hand, for the −*Z* direction, as this region is not the primary nucleation site, the morphology of the electrodes is relatively less influential on the growth of domains in the nucleation region. Therefore, for the −*Z* region, we opted for conventional rectangular comb-like electrodes.

**Figure 2:**
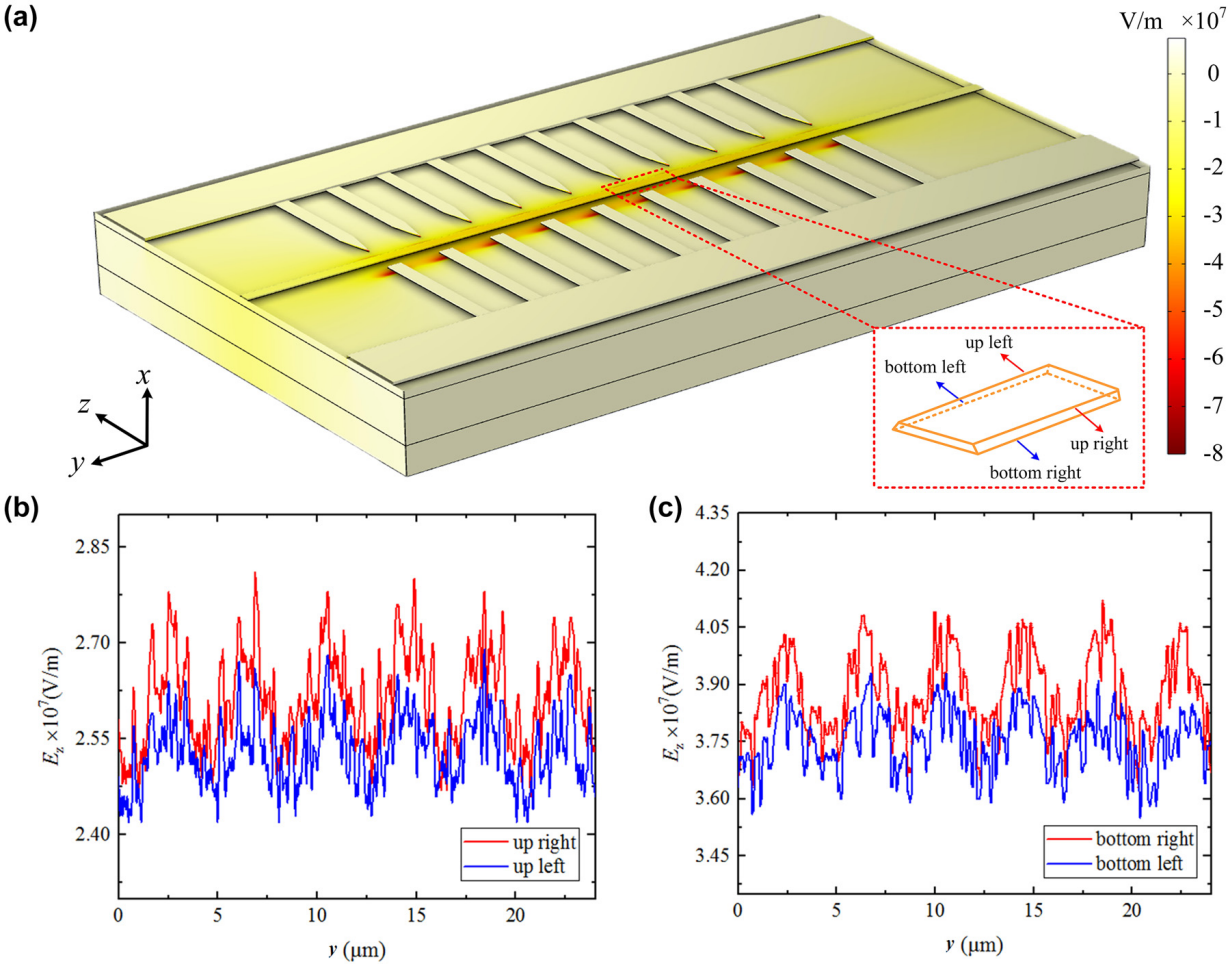
Simulated electric field distributions. (a) Distribution of the *z*-component of the electric field on the surface of the structure. (b) The plotted *z*-component of the electric field intensity at the upper two edges of the ridge waveguide. (c) The plotted *z*-component of the electric field intensity at the lower two edges of the ridge waveguide.

We used COMSOL to perform static electric field analysis on this structure, and the simulation parameters are: the voltage is 350 V, the spacing between the electrodes placed beside the ridge is 6 μm, the period of the electrode is 4.54 μm which is designed for SHG at around 1,550 nm, and the width of the electrode is 1 μm. The simulated results depicted in Figure 2(b) and (c) reveal an irregular distribution of the electric field. This uneven distribution is a result of the periodic arrangement of the electrodes, where the electric field strength is higher in areas closer to the electrode tips due to the tip effect. The presence of this tip effect presents challenges in achieving a uniform electric field distribution. Nevertheless, due to the high sensitivity of ferroelectric domain nucleation to electric field intensity, domain growth typically commences in regions with the most intense electric field, rather than simultaneously initiating across the entire uneven electric field region. Therefore, through experimental means, uniform domain reversal regions can be attained by capitalizing on the characteristics of the single-tip region of needle-like electrodes.

## 3 Experimental results

**Figure 3:**
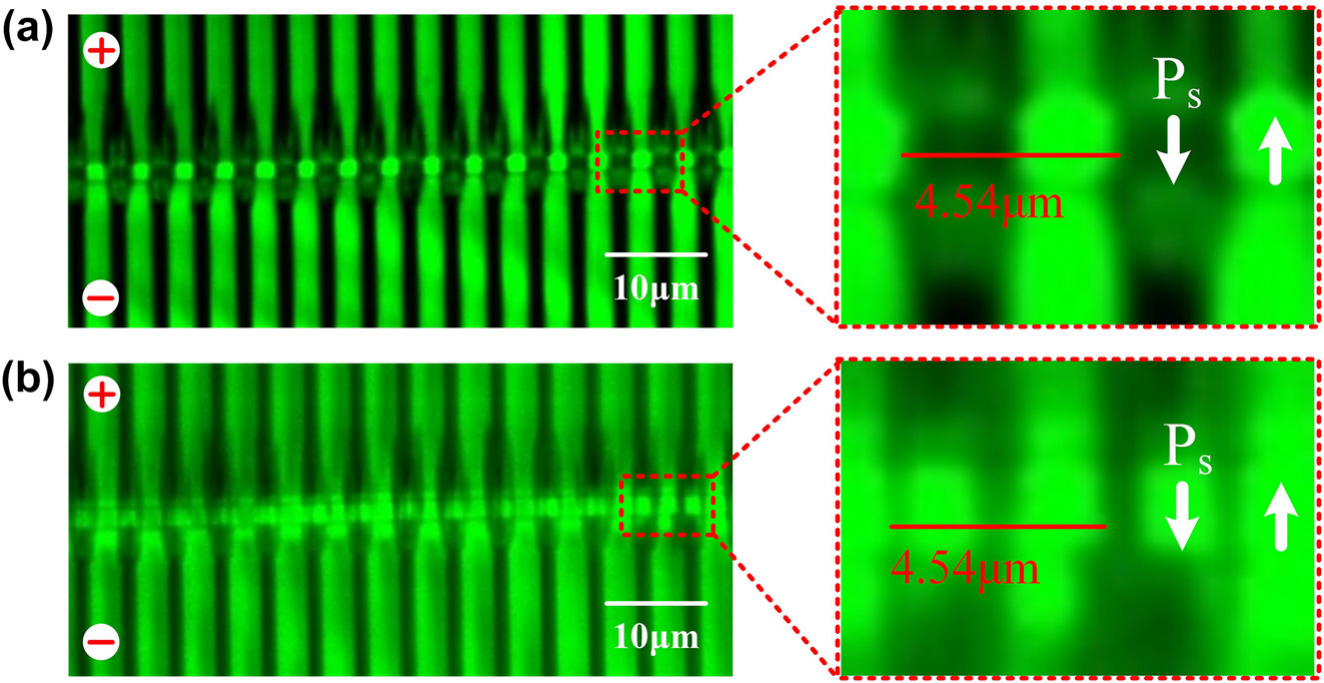
Images of the domain-inverted area revealed through SH confocal microscopy. The black areas represent the electrodes and the region in the middle is the waveguide area. (a) Confocal image beneath the surface of the ridge waveguide. (b) Confocal image when focused deeper inside the ridge waveguide.

After periodic poling, SH confocal microscope was used to characterize the inverted ferroelectric domains in the LNOI ridge waveguide, and the images are presented in Figure 3. Figure 3(a) presents the SH confocal image of the domain structure beneath the surface of the ridge, and one can see that the shape of the inverted domain in the ridge area is close to rectangular as expected. Figure 3(b) shows the SH confocal image when focused more deeper inside the ridge waveguide, which reveals a similar rectangular domain inversion.

**Figure 4:**
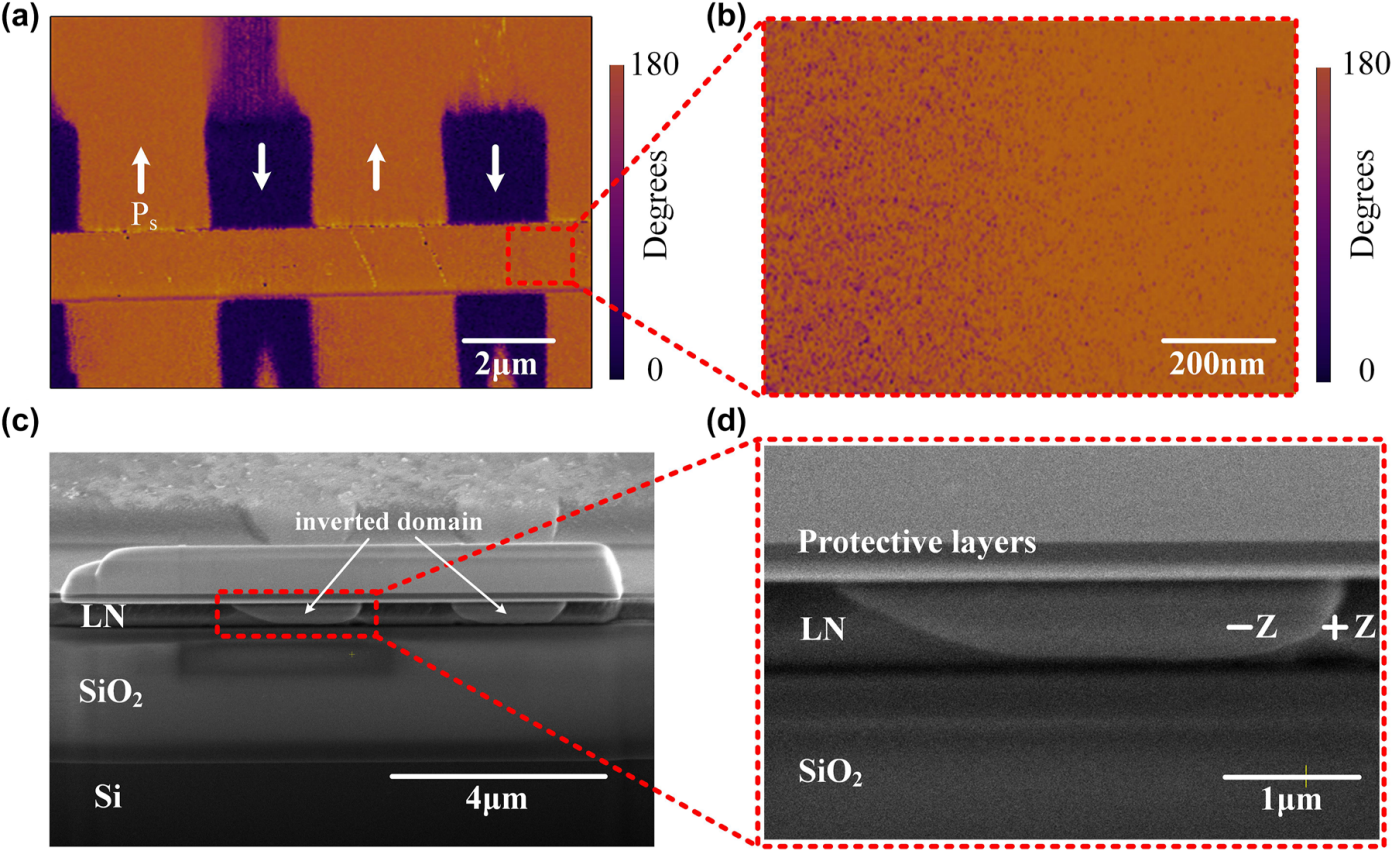
Inverted domain structure of the poled waveguide characterized by PFM, and SEM after chemical etching. (a) PFM phase image of the slab region of the ridge waveguide, which indicates the domain inversion. (b) Magnified view of the PFM phase image at the upper surface of the ridge, showing no signal of inverted domain was observed. (c) The SEM images of the sidewall of the waveguide, obtained through the precision cutting through FIB and subsequent RCA etching. The inverted domains are indicated by the arrows. (d) Magnified view of the domain structure inside the waveguide. A metal layer was deposited on the surface to serve as protective and conductive coating.

In order to investigate whether there has been complete polarization reversal in the depth of the waveguide region, we conducted piezoresponse force microscopy (PFM) tests. As shown in Figure 4(a) and (b), a strong signal for 180° inverted domain is observed on the slab region of the ridge waveguide, while no signal of inverted domain is detected at the upper surface of the ridge waveguide. To further investigate the inverted domain structure in the waveguide, we cut the sidewall of the ridge with focused ion beam (FIB), and revealed the domain structure by RCA (the same for the cleaning procedure) etching and SEM, as shown in Figure 4(c) and (d). The RCA etching process was conducted in a water bath at 60 °C for a duration of 8 h. We found that, from the upper surface of the ridge waveguide to a depth of about 70 nm, no domain inversion occurs. The failure of domain inversion near the upper surface of the ridge waveguide is closely related to the small electric field, which can be seen from the electric field distribution in Figure 2(b) and (c). The electric field at the upper surface of the ridge waveguide is about 7/10 that of the bottom, and due to which, the bottom of the ridge was domain inverted while the top part failed to invert. In order to achieve a thorough ferroelectric domain inversion along the depth direction within the ridge waveguide, it is essential to optimize the distribution of the electric field. Potential solutions to optimize this distribution include the use of thicker metal electrodes or the application of a dielectric layer, such as SiO_2_, on the wafer.

**Figure 5:**
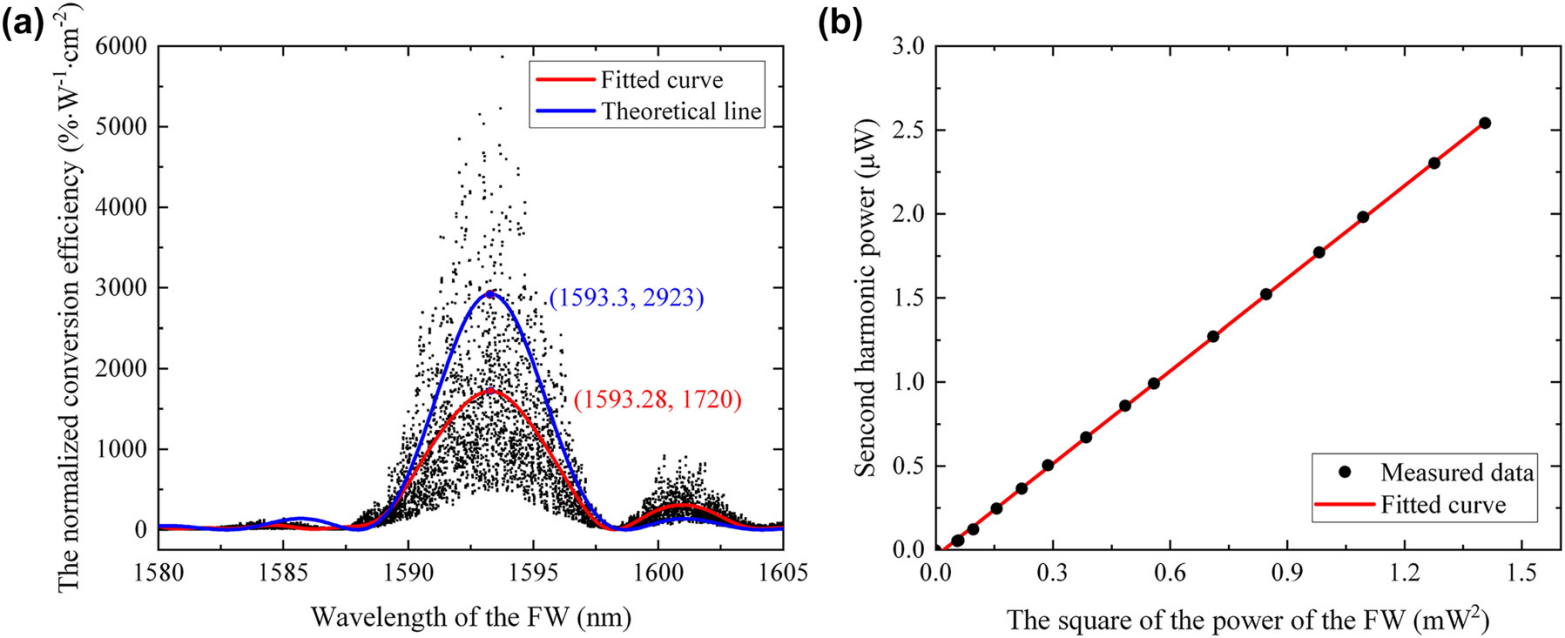
SHG characteristics of the fabricated PPLNOI (a) measured wavelength tuning curve for the SHG (black dot). The blue and red lines are the ideal and corrected transfer functions, respectively. (b) Quadratic power dependence of the SH wave on the FW.

The characteristics of the quasi-phase-matched nonlinear frequency conversion of the fabricated 4-mm-long periodically-poled LNOI ridge waveguide were investigated as well. A continuous-wave fiber laser (SANTECH, TSL-550), with the wavelength tuning range 1,480–1,630 nm, was used as the fundamental laser light source for the SHG test. The PPLNOI was designed for quasi-phase-matched type-0 SHG, and a polarization controller was used to ensure the excitation of the TE mode in the *x*-cut LNOI ridge waveguide. The infrared light was coupled into the waveguide with a tapered fiber for butt coupling, and a spherical mirror was used for output collection. To improve the coupling from the tapered fiber to the waveguide, 0.5 mm-long taper sections were fabricated at both ends of the straight waveguide, and the coupling efficiency was estimated to be 20 %. Behind the waveguide, a 45° low-pass filter was utilized to separate the SH and the fundamental wave (FW) for respectively measurement of the powers. Subsequently, a wavelength-dependent scan of the SHG conversion efficiency was conducted, as shown in Figure 5(a). It is important to note that, due to the polished end-facet of the waveguide, which introducing Fabry–Perot cavity effect, the scanning curve exhibited distinct oscillations [45], [46]. To mitigate this, data fitting was applied, resulting in a curve that closely followed the expected sinc^2^ function shape. At the quasi-phase-matched central wavelength of 1,593 nm, a normalized SHG conversion efficiency of 1,720 % W^−1^ cm^−2^ was achieved, which is about 60 % that of the theoretical value 2,923 % W^−1^ cm^−2^. The reduction of the conversion efficiency can be attributed to the two factors: one is the non-inverted ferroelectric domain near the upper surface of the ridge waveguide, and the second is the non-uniform film thickness of the LNOI wafer, which will reduce the effective interaction length of the periodical poled waveguide. The dependence of the output power of the generated SH wave on the square of the pumping power was plotted in Figure 5(b), which shows a linear relationship.

## 4 Conclusions

In this work, we have fabricated high-quality periodically poled *x*-cut LNOI waveguide for efficient quasi-phase-matched second harmonic generation. To avoid the selective etching effect of anti-parallel lithium niobate ferroelectric domains, which will introduce extra propagation loss to the ridge waveguide, periodical domain inversion was carried out after etching the ridge waveguide. To obtain the ideal rectangular shaped domain structure, we designed a comb-to-comb electrode which was placed away from the ridge waveguide, to provide an electrical field distribution similar in profile and amplitude at both sides of the ridge. The ridge waveguide was poled by applied high-voltage pulses, and the inverted domain structure was preliminarily characterized by SH confocal microscope, and PFM. In addition, the ridge waveguide was cut with FIB and was chemically etched to reveal the inverted domain structure inside the waveguide. We found that the inverted domain possessed nearly rectangular shape and part of the waveguide nearly the upper surface of the ridge waveguide failed to invert, which was consistent with the simulated electrical field distribution based on COMSOL. The quasi-phase-matched SHG of the fabricated PPLNOI ridge waveguide was characterized using a continuous-wave fibre laser in the communication L-band, and the normalized conversion efficiency of the 4-mm-long waveguide is measured to be 1,720 % W^−1^ cm^−2^, which is 60 % that of the theoretical one. The fabrication technique developed in this work will be beneficial for constructing efficient nonlinear photonic devices, especially for resonant devices, such as periodically poled whispering galley mode and race-track resonators.
